# Tracking the adoption of bread wheat varieties in Afghanistan using DNA fingerprinting

**DOI:** 10.1186/s12864-019-6015-4

**Published:** 2019-08-19

**Authors:** S. Dreisigacker, R. K. Sharma, E. Huttner, A. Karimov, M. Q. Obaidi, P. K. Singh, C. Sansaloni, R. Shrestha, K. Sonder, H.-J. Braun

**Affiliations:** 1 International Maize and Wheat Improvement Center (CIMMYT), Km45 Carretera Mexico-Veracruz, 56237 Texcoco, Mexico; 2 CIMMYT, #118, Lane-3, West of Bahristan Park, Kabul, Afghanistan; 3 Australian Center for International Agricultural Research (ACIAR), 38 Thynne Street, Bruce, ACT 2617 Australia; 4 CIMMYT-Turkey P.K. 39 Emek, 06511 Ankara, Turkey; 5 Agricultural Research Institute of Afghanistan (ARIA), Badam Bagh Agriculture Research Farm, Kabul, Afghanistan

**Keywords:** Afghanistan, DNA fingerprinting, Varietal adoption, Wheat

## Abstract

**Background:**

Wheat is the most important staple crop in Afghanistan and accounts for the main part of cereal production. However, wheat production has been unstable during the last decades and the country depends on seed imports. Wheat research in Afghanistan has emphasized releases of new, high-yielding and disease resistant varieties but rates of adoption of improved varieties are uncertain. We applied DNA fingerprinting to assess wheat varieties grown in farmers’ fields in four Afghan provinces.

**Results:**

Of 560 samples collected from farmers’ fields during the 2015–16 cropping season, 74% were identified as varieties released after 2000, which was more than the number reported by farmers and indicates the general prevalence of use of improved varieties, albeit unknowingly. At the same time, we found that local varieties and landraces have been replaced and were grown by 4% fewer farmers than previously reported. In 309 cases (58.5%), farmers correctly identified the variety they were growing, while in 219 cases (41.5%) farmers did not. We also established a reference library of released varieties, elite breeding lines, and Afghan landraces, which confirms the greater genetic diversity of the landraces and their potential importance as a genetic resource.

**Conclusions:**

Our study is the first in wheat to apply DNA fingerprinting at scale for an accurate assessment of wheat varietal adoption and our findings point up the importance of DNA fingerprinting for accuracy in varietal adoption studies.

**Electronic supplementary material:**

The online version of this article (10.1186/s12864-019-6015-4) contains supplementary material, which is available to authorized users.

## Background

About 54% of the population in Afghanistan still lives below the poverty line, with high rates of malnutrition (World Food Programme, https://www1.wfp.org/countries/afghanistan). Wheat is the primary staple food of most Afghanistan households [1]. The crop is grown on some 2.55 million hectares and more than 20 million rural people directly depend on it. On average, about 1.17 million hectares of irrigated wheat is grown each year and as much as 1.38 million hectares of rain-fed wheat. National wheat production has been highly erratic, ranging from 2.6 to 5.2 million tons of grain per year during the last decade, and Afghanistan depends on Iran, Kazakhstan and Pakistan to meet its domestic demand [2]. The Afghan Ministry of Agriculture, Irrigation and Livestock (MAIL) has estimated that Afghanistan would need about 7 million tons of wheat by 2022 to achieve self-sufficiency [3]. The more widespread use of improved seed and fertilizer on irrigated and rain-fed wheat fields, combined with better crop management, has been identified as a major development imperative. New cultivars have been shown to contribute to wheat yield gains of as much as 52% [4]. A study in Afghanistan estimated that use of improved wheat varieties alone could contribute raise yields as much as 33% under irrigated conditions and use of quality seed could enhance yield by a further 28% [5].

The last three decades of wheat research in Afghanistan has emphasized the release of new varieties. The country does not possess a national breeding program but rather evaluates genotypes bred elsewhere, continuously introducing them from several organizations and evaluating them in multi-location trials to identify the best-yielding and most disease resistant lines, which are finally released as varieties [6]. Since 2000, 40 new wheat varieties have been released, including some with the potential to produce around 6 t ha^− 1^ under irrigated conditions and up to 3.8 t ha^− 1^ under rain-fed conditions [2].

Measuring and understanding the adoption of improved crop varieties is challenging. Varietal adoption studies typically rely on the expert opinion of breeders, extension services, seed producers and suppliers, elicited responses from farmers through farm-level surveys, and morphological descriptors, among other sources. Gathering, assessing and collecting reliable information from such disparate sources entails huge complications and costs, as well as being subject to possible inaccuracies and inconsistencies.

Within the “Sustainable Wheat and Maize Production in Afghanistan” project led by the International Maize and Wheat Improvement Center (CIMMYT) in collaboration with MAIL with funding from the Australian Center of International Agricultural Research (ACIAR), primary data on the adoption of improved wheat varieties were recently derived through a farmer-level survey conducted in four Afghan provinces (Kabul, Herat, Balkh and Nangarhar) in 2016. The survey indicated that 88% of farmers who participated in on-farm demonstrations the previous year continued growing improved varieties in the seed-chain and shared seed with their relatives and neighbors, but inconsistent or incorrect use of variety names by farmers made it difficult to distinguish between traditional and new varieties.

Next generation sequencing technologies have become increasingly affordable and costs per sample are projected to continue decreasing [7, 8]. The emergence of DNA fingerprinting as a survey instrument provides the opportunity to use it to assess the accuracy of crop varietal identification and adoption in farmer surveys [9, 10]. In this study, we used DNA fingerprinting to (i) resolve uncertainties in the conclusions of a farmer survey regarding the adoption of improved wheat varieties in four Afghan provinces and (ii) establish a reference library of released varieties and landraces that will facilitate the identification of cultivars in farmers’ fields.

## Results

### SNP genotyping

A total of 56,422 DArTSeq® markers were scored across the 588 wheat varieties collected in Afghan farmers’ fields and the initially established reference library comprising 1019 entries. The SNP markers showed overall 13% missing values and 0.7% heterozygotes. Across marker loci, missing values ranged from 0 to 50% and heterozygote scores from 0 to 0.2%. Missing values across genotypes ranged from 3 to 88% and heterozygote scores from 0 to 14%. After filtering, our final data set included 1581 genotypes and 5203 SNP markers. The final collection of genotypes included 560 varieties from farmers’ fields, 965 varieties from the reference library, and 56 technical replicates (Additional file 1: Table S1). Most SNP markers could be mapped, 3033 SNP based on blastn of the DArTSeq® sequence tags to the Chinese Spring reference genome sequence (RefSeq.V1.0) and 4094 SNP based on a consensus map provided by the Genetic Analysis Service for Agriculture (SAGA) in Mexico, respectively. The numbers of SNPs per chromosome ranged between 34 on chromosome 4D to 434 on chromosome 2B.

### Identification of varieties in farmers’ field

Pairwise similarities between a random set of 28 entries (technical replicates), which were genotyped in triplicates were used to estimate the genotyping error rate. Paiwise identity-by-state (IBS) values of the technical replicates ranged from 0.991 to 1 with a mean of 0.998 (Additional file 1: Table S2). The initial IBS threshold was therefore set to 0.998. We subsequently applied this identity threshold on the pairwise IBS comparisons between the 560 farmer varieties and the reference library.

Although the initial identity threshold used to determine the farmer field samples was stringent, samples were in many cases found identical to more than one reference variety, partly because of inconsistencies in the reference library (see below).

We therefore applied the following assumptions during the field sample identification:

1. A sample was considered a farmer variety when its pairwise similarity was ≥0.998 to a single reference variety. We assumed the reference variety was correct.
2. A sample was considered a farmer variety when its pairwise similarity was ≥0.998 to several reference varieties, but to one reference variety that was represented more than once (duplicated because seed was obtain from multiple national sources). We assumed that if multiple seed sources of a reference variety were identical, the reference variety was correct and the most likely.
3. A sample was considered a farmer variety when its pairwise similarity was ≥0.998 to a single reference variety that was represented twice in the reference library (because seed was obtained from multiple national sources) but showed different genomic profiles. We assumed one seed source of the reference variety to be wrong.
4. A sample was considered not to be a farmer variety when its pairwise similarity was ≥0.998 to a reference varieties that was represented several times (duplicated ≥ four times) in the reference library, but each duplicate showed a different genomic profile. We assumed the seed sources of the reference varieties to be very uncertain and kept the farmer’s predicted name, when addtionally the farmer samples with the same name were highly identical among themselves.
5. A sample was considered not to be a farmer variety when no pairwise comparison ≥0.998 to any reference variety was observed, but varieties with the same name were highly identical among themselves. We assumed the reference variety was not included in the library and kept the farmer predicted name.
6. A sample was considered not to be a farmer variety when no pairwise comparison ≥0.991 (min. IBS threshold value) to any reference variety was observed and varieties with the same name had different genomic profiles. We assumed the reference variety was not included in the library and declared the farmer variety as ‘UNKNOWN’.

The identity of 528 (94.3%) varieties could be determined this way. In total 481 varieties (91.1%) showed pairwise similarities higher than the initial declared identity threshold of 0.998 to one or more reference varieties (Additional file 1: Table S3). For the remaining 47 varieties (8.9%), the initial threshold was relaxed to the minimum average pairwise similarity between technical replicates of 0.991. The identity of 32 farmer samples could not be determined (Additional file 1: Table S3). These varieties showed no pairwise similarity above the minimum threshold (0.991) to any reference variety (Additional file 1: Table S3).

**Fig. 1:**
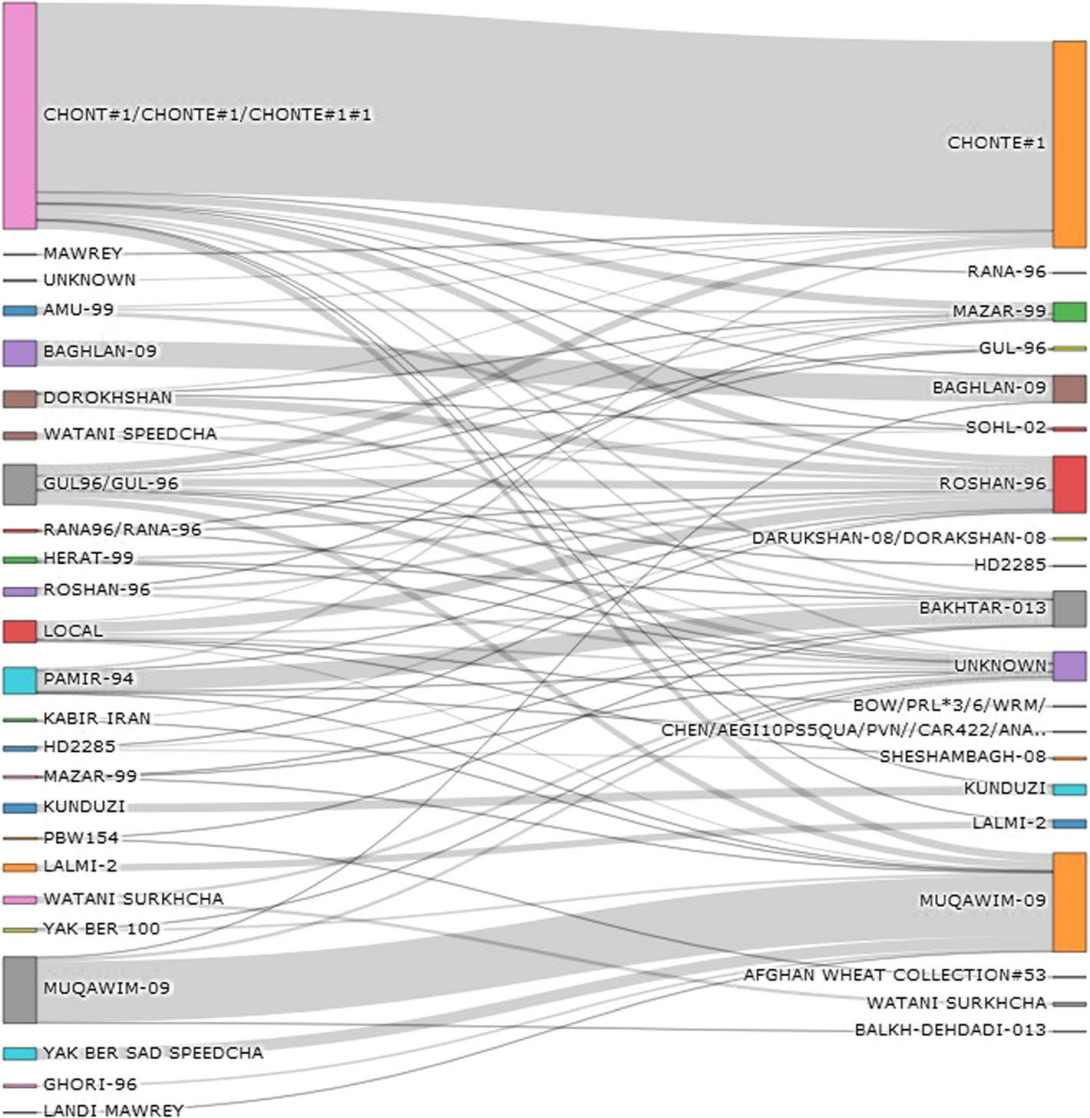
Sankey diagram capturing the relationship between wheat varieties reported by farmers (left) and wheat varieties determined using DNA fingerprinting (right). The colored bars indicate the percentage of total varieties observed by the farmers’ survey (24 varieties and the group ‘Local’ on the left) and by DNA fingerprinting (19 varieties and the group ‘Unknown’ on the right). The gray lines describe the individual genotypes and the relationship between the two methods

Farmers initially named 24 different varieties. DNA assessment, however, indicated that only 19 varieties were grown in the sampled provinces and season (Additional file 1: Table S3, Fig. 1). Fourteen varieties were officially released between 1993 and 2013 (Table 1). Nine varieties were direct releases from CIMMYT; others included CIMMYT lines as a parent. The farmer varieties determined as KUNDUZI, WATANI SURKHCHA, AFGHAN Wheat Collection #53, BOW/PRL*3/6/WRM, and CHEN/AEGI10PS5QUA or PVN//CAR422/ANA (the latter without complete pedigrees) were not on the official variety list. Nearly all field samples (93%) analyzed could be classified as improved varieties. The use of landraces was less than what farmers had claimed in the original survey.

**Table 1:** Released varieties in Afghanistan identified via DNA fingerprinting

Year	Name	Pedigree	Origin
2013	BAKHTAR-013	ISENGRAIN/ORNICAR	Cross made in another country
2013	BALKH-DEHDADI-013	PYN/BAU//MILAN	Cross made in another country, two CIMMYT parents
2010	CHONTE#1	SERI.1B*2/3/KAUZ*2/BOW//KAUZ/4/PBW343*2 KUKUNA	CIMMYT advanced line
2009	MUQAWIM-09	OASIS/SKAUZ//4*BCN/3/2*PASTOR	CIMMYT advanced line
2009	BAGHLAN-09	KIRITATI/SERI/RAYO	CIMMYT advanced line
2008	SHESHAMBAGH-08	SW89.5181/KAUZ	CIMMYT advanced line
2008	DORAKSHAN-08	CNDO/R143//ENTE/MEXI_2/3/AEGILOPS SQUARROSA (TAUS)/4/WEAVER/5/2*KAUZ	CIMMYT advanced line
2002	SOLH-02	OK82282//BOW/NKT	CIMMYT advanced line
2000	LALMI-2	BOBWHITE//MN	Cross made in another country, one CIMMYT parent
1999	MAZAR-99	PFAU/SERI-82//BOBWHITE	CIMMYT advanced line
1996	RANA-96	CA-8055/6/PATO/CALIDAD/3/7C//BB/CNO67/4/CALIDAD//CNO67/SONORA	Cross made in another country, one CIMMYT parent
1996	ROSHAN-96	BLOUDAN/3/BB/7C*2//Y50E/KAL*3	Cross made in another country, one CIMMYT parent
1996	GUL-96	ID 800994/3/KVZ/BUHO//KAL/BB or ID800994.W/VEE	CIMMYT advanced line
1993	HD2285	249/HD2160//HD2186 or HD1912–1592/hd1962E4870- K65XHD2160/HD2186	Cross made in another country, one CIMMYT parent

Both farmers and DNA fingerprinting found the varieties CHONTE-#1 (225 genotypes) and MUQAWIM-09 (108 genotypes) to be the most frequently grown (Table 2). Seven varieties (accounting for 50 samples) were not named by farmers (SOLH-02, SHESHAMBAGH-08, BALKH-DEHDADI-013, BAKHTAR-013, AFGHAN Wheat Collection #53, BOW/PRL*3/6/WRM, CHEN/AEGI10PS5QUA … /PVN//CAR422/ANA …). Of these, 47 samples were released after 2002 (Additional file 1: Table S3, Fig. 1), indicating that older varieties were unknowingly replaced by newer releases. Overall, 74% of the varieties were released after 2000, compared with the 65% reported by farmers. The percentage of local varieties and landraces was also only 8.8% according to the DNA fingerprinting, versus the 12.8% reported by farmers. In 309 cases (58.5%) farmers correctly identified the cultivar name, while in 219 cases (41.5%) farmer did not know what variety they were growing.

**Table 2:** Comparison of variety names reported by farmers and identified through DNA fingerprinting, for seed samples collected in Afghan farmers’ fields

#	Farmer reported variety name	# of entries	Variety name by DNA-fingerprinting	# of entries	Variety type
1	AMU-99	9			Released variety
2	BAGHLAN-09	28	BAGHLAN-09	30	Released variety
3	CHONT#1, CHONTE#1, CHONTE#1#1	247	CHONTE#1	225	Released variety
4	DOROKHSHAN	18	DARUKSHAN-08/DORAKSHAN-08	3	Released variety
5	GHORI-96	3		0	Released variety
6	GUL96, GUL-96	44	GUL-96	4	Released variety
7	HD 2285	5	HD2285	1	Released variety
8	HERAT-99	6			Released variety
9	KABIR IRAN	3			Released variety
10	KUNDUZI	9	KUNDUZI	10	variety
11	LALMI-2	8	LALMI-2	9	Released variety
12	LANDI MAWREY	1			Landrace
13	MAWREY	1			Landrace
14	MAZAR-99	3	MAZAR-99	20	Released variety
15	MUQAWIM-09	72	MUQAWIM-09	108	Released variety
16	PAMIR-94	29			Released variety
17	PBW154	2			Released variety
18	RANA96, RANA-96,	3	RANA-96	1	Released variety
19	ROSHAN-96	9	ROSHAN-96	63	Released variety
20	WATANI SPEEDCHA	9		0	Landrace
21	WATANI SURKHCHA	8	WATANI SURKHCHA	4	Landrace
22	YAK BER 100	4			Landrace
23	YAK BER SAD SPEEDCHA	13			Landrace
24			SHESHAMBAGH-08	3	Released variety
25			BALKH-DEHDADI-013	1	Released variety
26			SOLH-02	4	Released variety
27			BAKHTAR-013	39	Released variety
28	LOCAL/UNKNOWN	26	UNKNOWN	33	–
29			BOW/PRL*3/6/WRM/	1	Breeding line
30			CHEN/AEGI10PS5QUA,PVN//CAR422/ANA	1	Breeding line
31			AFGHAN Wheat Collection#53	1	Landrace

Regarding the distribution of farmer varieties, 10 were grown in the Herat Province, 9 in Kabul Province and 7 each in Balkh and Nangarhar Provinces (Table 3). Some varieties or landraces were only grown in only one province; e.g., BAGHLAN-09, DARUKSHAN-08/DORAKSHAN-08, LALMI-2, SHESHAMBAGH-08, KUNDUZI and WATANI SURKHCHA. The most popular varieties (CHONTE#1 and MUQAWIM-09) were grown in all four provinces.

**Table 3:** Farmer varieties identified through DNA fingerprinting grown in four Afghan provinces

Varieties assessed by DNA fingerprinting	Type	Province (District)				Total
		Herat (Gozara)	Nangarhar (Surkh Rod)	Kabul (Bagrami)	Balkh (Dehdadi, Nahre Shahi)	
BAKHTAR-013	released variety	13		26		39
BALKH-DEHDADI-013	released variety		1			1
CHONTE#1	released variety	5	72	67	81	225
MUQAWIM-09	released variety	58	22	5	23	108
BAGHLAN-09	released variety		30			30
SHESHAMBAGH-08	released variety	3				3
DARUKSHAN-08/DORAKSHAN-08	released variety			3		3
SOLH-02	released variety	1		3		4
LALMI-2	released variety		9			9
MAZAR-99	released variety	9	4	5	2	20
RANA-96	released variety				1	1
ROSHAN-96	released variety	40	1	17	5	63
GUL-96	released variety	1		3		4
HD2285	released variety	1				1
KUNDUZI	variety				10	10
BOW/PRL*3/6/WRM …	breeding line	1				1
CHEN/AEGI10PS5QUA, PVN//CAR422/ANA	breeding line			1		1
WATANI SURKHCHA	landrace				4	4
AFGHAN Wheat Collection#53	landrace	1				1
Unknown	likely landraces	13	2	9	8	32
Total		146	141	139	134	560

### Hierarchical and model-based clustering

**Fig. 2:**
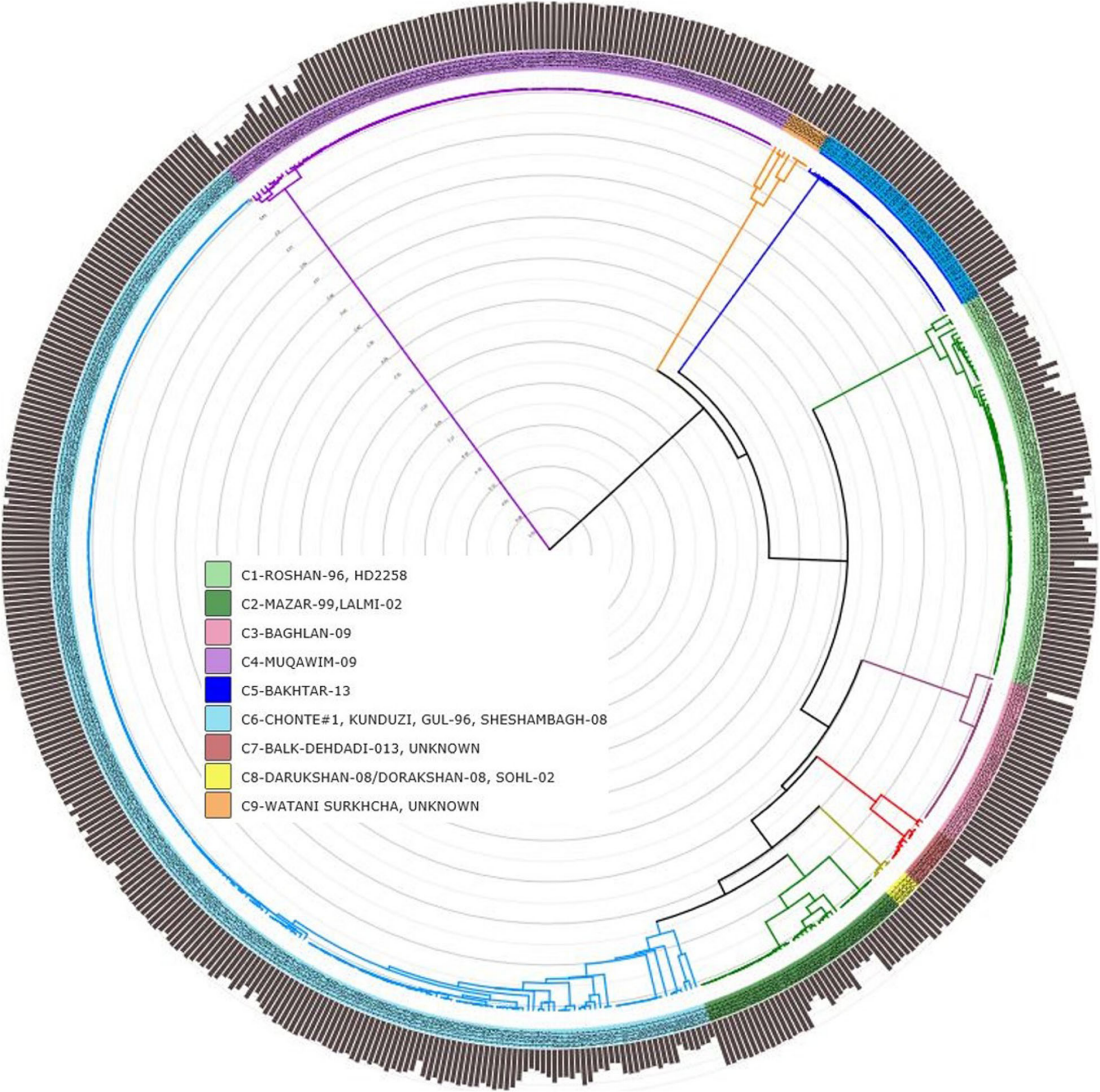
Circular phylogram of farmer varieties using IBS based on 5203 SNP markers as a similarity matrix and the Ward method for clustering. Major clusters are colored according to the legend. The outer bars represent the ancestry coefficient derived from model-based clustering ranging from 0 to 1

To visualize the observed identities and confirm the close genetic relationship between the samples declared to be the same variety, model-based clustering and hierarchical analysis were performed for the 560 genotypes from farmers’ fields. For population structure analyses, 1 to 15 clusters were tested based on the cross-entropy criterion. The cross-entropy curve exhibited a plateau at K = 9 (Additional file 2). An ancestry-coefficients matrix was therefore generated, assuming nine major genotype groups. Each genotype was assigned to its respective group when the ancestry coefficient was above 0.5 (Additional file 3). The overall grouping was highly correlated with the re-identified variety names using the IBS matrix (Additional file 1: Table S3, Fig. 2), while some varieties grouped together. Group 1 included all genotypes identified to be ROSHAN-96, except one with a percent ancestor contribution of 0.528 to Group 6. Group 2 consisted of all genotypes identified to be MAZAR-99 and LALMI-2. Group 3 included all genotypes identified to be BAGHLAN-09. Group 4 contained all genotypes that were identified as MUQAWIM-09, except two with a percent ancestor contribution of 0.522 and 0.604 to Group 2 and Group 6, respectively. Group 5 included all genotypes identified to be BAKHTAR-013. Group 6 was the largest group and consisted of genotypes with three determined varieties (CHONTE #1, KUNDUZI, and GUL-96). Group 7 contained eight genotypes whose’ identity could not be determined with DNA fingerprinting. Group 8 included the identified varieties DARUKSHAN-08/DORAKSHAN-08 and SOLH-02 and Group 9 the identified landrace WATANI SURKHCHA and additional varieties of unknown identity. Overall, 45 (8.0%) genotypes could not be assigned to any of the nine groups, indicating admixed genotypes.

Hierarchical cluster analyses using the Ward clustering method confirmed the nine major groups from population structure analyses (Additional file 1: Table S3, Fig. 2). Only 15 genotypes clustered differently. The 45 genotypes that could not be assigned to any of the nine groups in population structure analyses grouped to different clusters in hierarchical clustering but often formed a small sub-cluster. Sub-clusters were also built when two or more varieties formed one cluster e.g. Cluster 2 or Cluster 6 (Fig. 2).

### Trait-based marker results

To complement the DArTSeq® results, farmer varieties were additionally evaluated with a set of informative markers associated with genes falling in the category of rust resistance and crop development (Additional file 1: Table S4). Trait-based markers results were complementarily aligned to the determined farmer varieties (Additional file 1: Table S5). Variety BAGHLAN-09 carried two adult-plant resistance genes (*Lr68* and *Sr2*). Most varieties carried one of the major alleles reducing plant height, *Rht-B1b* or *Rht-D1b* (dwarf phenotype), except for variety RANA-96 and the landrace WATANI SURKHCHA. Only variety GUL-96 was identified to be a true winter type. All other cultivars had one or two spring alleles at the most relevant vernalization loci. All culivars were photoperiod insensitive except the landrace WATANI SURKHCHA.

### Establishment and characterization of the reference library

The initial reference library included 176 varieties from the Afghan Wheat Collection and 843 varieties representing local landraces, varieties released in Afghanistan and other countries and additional elite breeding lines internationally distributed by the CIMMYT spring bread wheat and winter wheat breeding programs. Based on the IBS matrix and additional crosschecks of the varieties’ pedigrees in public and institutional databases, the reference library was reduced to 761 entries. Excluded were identical varieties (varieties with the same name, same pedigree and same genomic profile) and varieties with incomplete names or pedigrees. Despite these efforts, substantial inconsistencies somewhat impair the overall utility of this reference library. After cleaning, 13.7% of the varieties had the same name and pedigree, but distinct genomic profiles. This measure did not include breeding lines with the same name and pedigree but distinct genetic profiles for which multiple sister lines exist. The measure also did not consider reference varieties genetically different, but with the same landraces name due to probable higher genetic heterogeneity of landraces. On the other hand, 7.2% of the varieties had the same genomic profile but different names and pedigrees. Thus, information for 20.9% of the varieties in the reference library was clearly inconsistent.

**Fig. 3:**
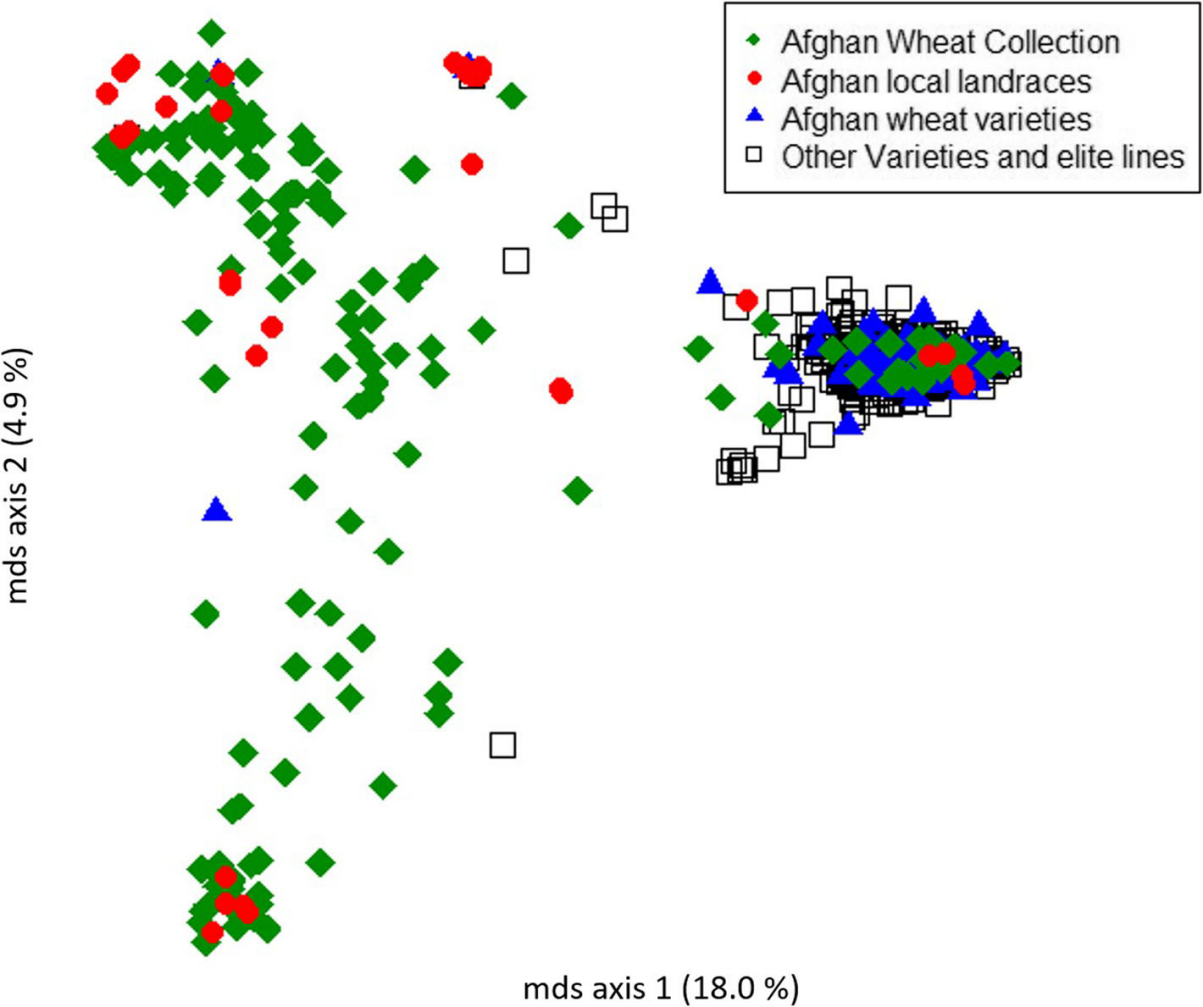
Multidimentional scaling of the established Afghan reference library based on Modified Rodgers’ distance using 5203 SNP markers

The reduced reference library was classified into four groups (Additional file 1: Table S6): (i) varieties from the Afghan wheat collection (152), (ii) Afghan local landraces (36), (iii) Afghan released varieties (125), and (iv) other varieties and advanced breeding lines (448). Using multidimensional scaling based on MRD distance, all varieties and breeding lines (except three) built one close group, while most of the varieties in the Afghan wheat collection and the Afghan local landraces grouped apart (Fig. 3). Afghan released varieties fell within almost all the dimensional space that was covered by other varieties and breeding lines, showing that the varieties originated from different germplasm sources. Diversity indices for the four germplasm groups are shown in Additional file 1: Table S7. The indices support the multi-dimensional scaling results, with the Afghan wheat collection and Afghan local landraces presenting the greatest genetic diversity. Afghan released varieties represented the lowest genetic diversity, which is expected because the varieties represent only a subset of the fourth group of internationally released varieties and advanced breeding lines.

## Discussion

Our study is the first in wheat to apply DNA fingerprinting at scale to get an accurate assessment of wheat variety adoption in Afghanistan during the main cropping season in 2015/2016. Using DNA fingerprinting, we were able to identify 94.4% of the varieties collected in farmers’ fields in four Afghan provinces. Thus, the approach represents a reliable way to resolve uncertainties in studies to assess the adoption of improved wheat varieties.

To date, DNA fingerprinting in plant breeding has mainly been used to enforce intellectual property rights, including plant variety protection in more developed economies. The International Union for the Protection of New Varieties of Plants (UPOV) still relies on the phenotypic assessment of Distinctness, Uniformity and Stability (DUS), but has considered DNA fingerprinting as new method, while workable solutions for each crop such as technical rules, standard statistical methodologies and established thresholds are still required [11]. We suggest that in developing countries DNA fingerprinting can support the collection of accurate variety-specific identification data to study adoption rates. Crop improvement is a major activity of CGIAR centers and thousands of new varieties are developed annually to provide higher yield, better nutritional content or increased resistance to diseases or other biotic and abiotic stresses in diverse agro-ecological settings. Accurate information on crop varieties is crucial to determine the extent of farmer adoption and evaluate the performance of agricultural development programs. The use of DNA fingerprinting to address this objective in wheat and other crops is still limited with only a few reported pilot studies. Rabbi et al. [10] were the first using genotyping-by-sequencing as an alternative method to track released cassava varieties in farmers’ fields. Many synonymous or homonymous clone names in cassava made it difficult to track released varieties by relying on names only. In total, 88% of the 917 cassava accessions were matched to specific released varieties or landraces in the reference library. Kosmowski et al. [12] tested the effectiveness of three household-based survey methods of identifying sweet potato varietal adoption against DNA fingerprinting. All other methods were found to be less accurate than the DNA fingerprinting benchmark. Similar to the study in cassava, variety names given by farmers provided inconsistent varietal identities. A comprehensive comparison of different approaches to collect variety-specific adoption data was also published by Maredia et al. [9] for cassava and beans. The authors compared six different approaches including farmer and expert elicitation. Each method provided different estimates of adoption rates, but no method could be specifically recommended. All methods underestimated the adoption of improved varieties and misclassified improved and local varieties. The authors pointed out that DNA fingerprinting was the only credible method.

Wheat research in Afghanistan has emphasized the release of new, improved varieties supported by various agricultural development programs; e.g., the ACIAR ‘Wheat and maize projects in Afghanistan’ and few others implemented by the Food and Agriculture Organization (FAO) of the United Nations, the International Centre of Agricultural Research in Dry Areas (ICARDA) or French Cooperation. According to the CIMMYT Wheat Atlas (http://wheatatlas.org/varieties, accessed on November 1, 2018), and Sharma and Nang [2], 40 wheat varieties have been released since 2000 (Additional file 1: Table S8). Our DNA assessments of seed collected during the 2015–16 main cropping season showed a general prevalence of farmers growing new varieties across 560 surveyed farms. The number of farmers using improved varieties was higher than the number reported in the 2016 farmer survey. Thus, similar to previous studies, the farmer-level survey performed in 2016 in the same project underestimated the adoption of improved varieties. Across the four provinces, 75.4% of the farmers were growing post-2000 released varieties, even though 59.5% of the farmers were growing the two most popular varieties released in 2009 and 2010, MUQAWIM-09 and CHONTE#1, respectively. The most recent varieties grown by farmers were released in 2013 (BALKH-DEHDADI-013 and BAKHTAR-013). Based on current trends, varieties released more recently have entered the seed multiplication system and it can be assumed that farmer adoption will become significant when seed becomes available. The total volume of certified seed available in Afghanistan has declined during the last decade due in part to the existence of an artificial seed market fueled by inconsistent, foreign aid supported subsidies [2]. MAIL has taken several recent steps to foster the emergence of a demand-driven seed industry; e.g., allowing seed enterprises to produce truthfully labeled seed. In 2017/18, improved varieties released since 2002 accounted for about 70% of total certified seed produced. It is expected that post 2012 varieties will dominate the certified seed system within the next 5 years.

The two most popular varieties MUQAWIM-09 and CHONTE#1 were grown in all four provinces; however, CHONTE#1 was least frequent in the Herat province, where MUQAWIM-09 was most frequent. On the other hand, MUQAWIM-09 was least frequent in the Kabul province (Table 3). There are two main seed sources for Afghan wheat farmers, one from government supplies or agencies like CIMMYT and second source is self-saved or from neighbors, friends or relatives etc. Because the governmental supply has been insufficient, a great majority of farmers rely mainly on self-saved seed. In Herat, around 312 tons of certified seed of MUQAWIM-09 was produced, in 2014–2015 while none of CHONTE#1. In addition, CIMMYT distributed seed of MUQAWIM-09 to 101 farmers the same year, which explains why MUQAWIM-09 was the most dominant variety in this province. In Kabul, certified seed of MUQAWIM-09 and CHONTE#1 accounted for 13 and 5% of the total certified seed production, respectively. Additionally, CIMMYT distributed CHONTE#1 to 120 farmers in the Kabul province in 2014–15. The preference for CHONTE#1 in the Kabul province must therefore have mainly been derived from spillover of the distributed seed in the previous year.

Overall 4% fewer local varieties and landraces than reported by the farmers were grown in 2015/16, indicating that older varieties had been unknowingly replaced. Several previous survey studies have reported the consensus belief of farmers in Afghanistan that having access to and planting improved seed varieties will be advantageous [13–15]. The motivations of farmers surveyed for planting improved seed were mainly higher yields and better insect and disease resistance [13]. The most recent and heightened risk to Afghanistan’s wheat production has come from the Ug99 stem rust race. It was estimated that the disease could reduce the country’s annual wheat production by as much as 20% [16]. Migration trends from Iran, where a Ug99 stem rust outbreak was reported in 2007, to Afghanistan, coupled with the presence of dangerous new races of yellow rust, Afghanistan’s major wheat disease, have alarmed the Afghan national research system. Extensive national rust screening nurseries are being conducted and steps have been taken to remove susceptible varieties from Afghanistan’s seed chain. Our DNA assessment showed that local varieties and landraces as well as rust susceptible varieties such as GHORI-96, GUL-96, and PAMIR-94 have mainly been replaced by the variety MUQAWIM-09. Both popular varieties MUQAWIM-09 and CHONTE#1 are resistant to Ug99 [17], although CHONTE#1 has been found to be susceptible in southern Pakistan to the new Kiran-virulence stem rust race (16). This emphasizes the need to maintain extensive rust monitoring and to continuously track wheat virulence development in the country. DNA fingerprinting assessments such as the present one can underpin wheat breeding, varietal release policies and especially the continual replacement of old varieties with new, high-yielding and disease resistant ones, all of which is crucial for food security in a country such as Afghanistan.

Our study was not without limitations. First, the sampling strategy for collections in the farmers’ fields was restricted. Spikes of five representative plants were sampled, which might have been too small for precise identification for farmer varieties. Although our results determined a number of admixtures within farmer varieties (8%), heterogeneity in farmers’ fields is likely underestimated, especially because heterogenous varieties such as local landraces were expected to be grown and were still grown, albeit at a low rate. Heterozygote SNPs were ignored during SNP data filtering because of the risk of miscalling heterozygotes based on the low sequencing depths of the genotyping technology used. This step reduced the genotyping error rate (data not published) but has most likely led to additional undetected heterogeneity. Second, the reference library suffers from inconsistencies. The DNA assessment of varieties can only be as good as the quality of the reference library. Inconsistencies were partly expected, considering the difficulties of compiling the library in a country with severe social and political unrest and insecurity and limited local seed stocks. Varieties in the library with synonymous names showed different genetic profiles and vice versa. In some cases, varieties collected in farmers’ field showed similarities to more than one reference variety, necessitating additional assumptions for varietal determination. To gain confidence in our results, we subsequently compared the farmer varieties with seed stocks of varieties in CIMMYT’s germplasm bank, genotyped with the same SNP platform (data not shown). These additional pairwise comparisons confirmed the identity of farmer varieties CHONTE#1 (the most popular), SOLH-02, MAZAR-99, ROSHAN-96 and LALMI-2.

Our reference library included 188 Afghan landraces from the Kihara Afghan wheat landrace collection and local sources. The landraces showed greater genetic diversity than released varieties and elite breeding lines in the library. This finding confirms the importance of the Afghan landraces as genetic resources and the imperative of maintaining representative ex-situ collections. The landraces have been evaluated for several foliar diseases and several have multiple disease resistance enhancing their utility for wheat breeding programs. The complete Kihara Afghan wheat landrace collection was also recently genetically and phenotypically evaluated for use in breeding [18–21]. Manickavelu et al. [19] conducted a genome-wide association study on 352 Afghan wheat landraces and revealed new resistance loci for wheat stripe rust. Kondou et al. [22] and Manickavelu et al. [20] evaluated the collection for grain elements.

## Conclusions

There is increasing evidence across a range of crops, now including wheat, that DNA fingerprinting is a potent tool to assess the adoption of modern varieties. DNA assessment of farmer varieties in Afghanistan could resolve the uncertainty of release, dissemination and adoption of improved, disease resistant, high performing wheat varieties. Our results will stimulate a wider use of DNA fingerprinting in adoption and impact assessments and suggest that estimating the adoption of improved varieties with methods based on farmer self-reports are less reliable. As the cost of DNA fingerprinting declines, the cost of conducting a survey will become more affordable. Our findings also point up the value of DNA fingerprinting in adoption studies to ensure the accuracy of socio-economic research in agriculture and the relevance of associated policy recommendations.

## Methods

### Wheat seed samples from farmers’ field

**Fig. 4:**
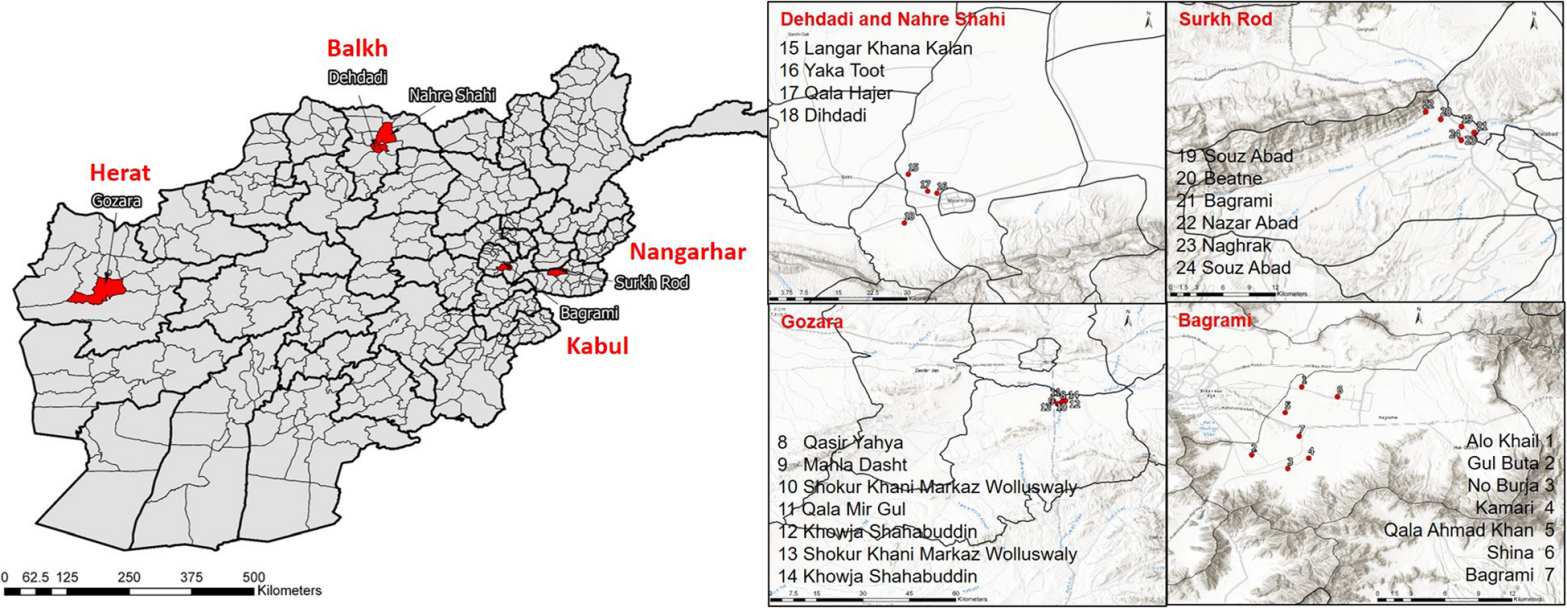
Geographical presentation of the sampling locations. Provinces and districts are indicated on the left and villages within districts on the right. Seed sample collections for DNA fingerprinting were made in farmers’ wheat fields in the proximity of the villages

We collected seed samples of 588 wheat varieties in farmers’ fields in four provinces in three agro-ecological zones of Afghanistan. The four provinces were (i) Herat in West Afghanistan (Gozara District), where crops are grown with irrigation from surrounding rivers; (ii) Nangarhar (Surkh Rod District) and (iii) Kabul (Bagrami District) in East and Central Afghanistan, where double or triple cropping using rich river water is predominant; and (iv) Balkh (Nahre Shahi and Dehdadi District), in the major farm area in the northern Afghanistan (Fig. 4). CIMMYT staff collected the seed during the main growing season in 2015/2016 in 24 villages located within the four provinces and districts (Fig. 4.). The villages were in the vicinity of CIMMYT established hubs to test and demonstrate improved varieties and agronomic practices. One third of selected farmers in the villages had received material from the hubs while two thirds were located in the same area but had not been involved with the hubs. Farmers were interviewed and asked for permission to collect seed samples. Each of the 588 seed samples were obtained from spikes of five representative wheat plants dispersed within one randomly selected wheat field in the proximity of the villages. For DNA fingerprinting all grain was air dried to constant weight, mixed and a random 3.5 g sample of grain was selected.

### Reference library

To determine the wheat varieties actually grown by farmers, we compiled a comprehensive wheat reference library comprising 1019 entries. The reference library included primarily released varieties and landraces from the country, but also other varieties and advanced breeding lines from diverse seed sources. The latter were included to compare the genetic diversity of the local germplasm with a broader set of wheat materials. A subset of 176 entries was derived from the Kihara Afghan wheat landrace collection housed in the Kihara Institute for Biological Research in Japan [18]. Seed of the remaining 843 entries was obtained directly from the Agricultural Research Institute of Afghanistan (ARIA), including varieties officially released in Afghanistan and neighboring countries, local landraces and CIMMYT advanced breeding lines distributed via the International Wheat Improvement Network (IWIN). In many cases, seed of the same variety from multiple sources was included. All reference varieties were grown at the ARIA Darul Aman research farm in Kabul during the 2015–2016 cropping season. Representative spikes of several plants were collected, the grain was air-dried, mixed, and a random 3.5 g sample of grain was selected. All samples were transported to CIMMYT headquarter in Mexico. Among 25 to 30 seeds of each sample were grown in the greenhouse in Mexico during 2016/2017 for seed multiplication and storage in the CIMMYT wheat germplasm bank.

### DNA extraction and genotyping

During seed multiplication, a small leaf sample of each plant per entry was collected and bulked. DNA was extracted from the ground leaves using a modified CTAB procedure described in Dreisigacker et al. [23]. All entries were fingerprinted using the DArTSeq® technology at the SAGA in Mexico. DArTSeq® is based on a complexity reduction method including two enzymes (*PstI* and *HpaII*) to generate a genome representation of the selected set of samples. *PstI*-RE site specific adapter was tagged with 96 different barcodes enabling multiplexing a plate of DNA samples to run within a single lane on Illumina HiSeq2500 instrument (Illumina Inc., San Diego, CA). The successful amplified fragments were sequenced up to 77 bases, generating approximately 500,000 unique reads per sample. Thereafter the FASTQ files (full reads of 77 bp) were quality filtered. A proprietary analytical pipeline developed by DArT P/L was used to generate allele calls for SNPs. To estimate the genotyping error rate, a random set of 29 entries was genotyped in triplicates (technical replicates). Farmer varieties were additionally evaluated with a set of informative markers associated with genes related to rust resistance and crop development (Additional file 1: Table S4). Informative markers were scored using Sequence-Tagged Sites (STS) markers and Kompetetive Allele Specific PCR (KASP) designs and protocols [23]. Briefly, the polymerase chain reaction (PCR) assay reaction mixture in single 10 μl reactions used to amplify all STS primers contained final concentrations of 1× Buffer with Green Dye (Promega Corp., US), 200 μM deoxynucleotide triphosphates (dNTPs), 1.2 mM magnesium chloride (MgCl_2_), 0.25 μM of each primer, 1 U of DNA polymerase (GoTaq®Flexi, Promega Corp., Cat. # M8295) and 50 ng of DNA template. The PCR profile was 94 °C for 2 min followed by 30 cycles of 94 °C for 1 min, 54 °C to 60 °C for 2 min (dependent on the primer), and 72 °C for 2 min. The amplified products were separated on 1.2% agarose gels in tris-acetate/ethylene-diamine-tetraacetic acid (TAE) buffer. The KASP were scored using KASP reagents (https://www.biosearchtech.com) in reactions containing 2.5 ml water, 2.5 ml 2 × KASPar Reaction mix, 0.07 ml assay mix and 50 ng of dried DNA with a PCR profile of 94 °C for 15 min activation time followed by 20 cycles of 94 °C for 10 s, 57 °C for 5 s and 72 °C for 10 s and followed by 18 cycles of 94 °C for 10 s, 57 °C for 20 s, and 72 °C for 40 s. Fluorescence was read as an end point reading at 25 °C.

### Statistical analyses

SNP filtering was performed. Because of the high risk of miscalling heterozygote SNPs, due to the low sequencing depth of our genotyping platform, all heterozygote scores were ignored and converted to missing data to mimize genotyping errors. Markers and genotypes with ≥20% missing data were excluded. Furthermore, markers with an allele frequencies less than 0.01 (MAF < 0.01) were removed. Marker imputation was not considered.

An identity matrix was computed by pairwise comparison of genotypes across all SNP sites. IBS was computed with the following equation:

$$ {IBS}_{ij}=\frac{\sum_{x=1}^n\left({allele}_{ix}=={allele}_{jx}\right)}{n}, $$

where IBS for a given pair of genotypes *i* and *j*, *allele*_*ix*_ and *allele*_*jx*_ are the alleles at the *x*^*th*^ SNP, respectively, *n* the number of total SNP sites, and the == sign represent an exact successful match between two alleles. IBS thus compares SNP profiles for any two individuals, in which individuals are observed to have 0, 1, or 2 alleles in common at any given SNP site throughout the genome. The IBS matrix was computed using the R-package GenABEL version 1.8–0 [24].

A genotyping error rate or threshold IBS value was determined to declare whether two genotypes are identical. This value was empirically determined from the distribution of average pairwise IBS values between the technical replicated DNA (29 varieties genotyped in triplicate). The overall arithmetic mean of IBS values among technical replicates was declared as initial threshold. This threshold was then applied to the complete IBS matrix to compare the varieties from farmers’ fields with the varieties in the reference library and to determine variety identity.

Whereas pairwise comparisons of genotypes provide an absolute percent of similarity between individuals, clustering can provide an additional independent support. While it is difficult to interpret clustering results to identify identical genotypes, it can outline obvious outliers and misclassified individuals [10]. A Ward’s minimum variance hierarchical cluster dendrogram was therefore built from the IBS similarity matrix. Furthermore structure population inference algorithms were performed, choosing different number of clusters and showing admixture coefficients. Choosing the number of clusters was based on cross-validation and on an information theoretic measure, the cross-entropy criterion. The approach was used additionally to identify the probable ancestries of the entries and mixtures. Hierarchical and model-based clustering were performed in R using the packages, ‘ape’ version 5.2 and ‘LEA’ version 2.0.0, respectively.

Standard genetic diversity measures were estimated in the reference library. The reference library was divided into four germplasm groups: (i) accessions from the Kihara Afghan wheat collection, (ii) Afghan local landraces, (iii) varieties officially released in Afghanistan and (iv) other varieties and CIMMYT elite breeding lines. Modified Rogers’ distance (MRD) [25] was calculated among all possible pairs of genotypes as a basis for applying multivariate methods, because it represents a Euclidean distance. Multi-dimensional scaling [26] was applied to represent visually the patterns of genetic variability. Genetic diversity indices among the defined germplasm groups included an estimate of expected heterozygosity, which accounts for the richness and evenness of alleles, the Shannon diversity index and the mean of the MRD distances within each group. All genetic diversity analyses were implemented in base R.

## Additional files

Additional file 1: **Table S1.** Initial list and source of 1581 entries used for data analyses, including 965 reference varities, 650 farmers’ samples and 56 technical replicates. **Table S2.** Pairwise identity by state (IBS) similarities among technical replicates. **Table S3.** Predicted and with DNA fingerprinting re-identified cultivars from farmers field in Afghanistan**. Table S4.** Informative markers applied across collected Afghan field samples. **Table S5.** Presence of genes in identified wheat varieties in Afghan farmers fields. **Table S6.** Established Afghan reference library. **Table S7.** Diversity indicies of germplasm groups represented in the established Afghan reference library. **Table S8.** Varieties released in Afghanistan since 2000. (XLSX 189 kb)

Additional file 2: Cross-entropy plot for the SSC when the number of clusters ranges between K = 1–15. (JPG 25 kb)

Additional file 3: Diagram of groups derived from model based clustering of wheat varieties reported by farmers. (JPG 231 kb)

## Data Availability

The genotypic data used or analyzed in the current study are available in the CIMMYT Research Data Repository (https://data.cimmyt.org/dataset.xhtml?persistentId=hdl:11529/10548167).

## Abbreviations

ACIAR: Australian Center of International Agricultural Research
ARIA: Agricultural Research Institute of Afghanistan,
CIMMYT: International Maize and Wheat Improvement Center
CTAB: Cetyl trimethylammonium bromide
IBS: Identity-by-state
IWIN: International Wheat Improvement Network
KASP: Kompetetive Allele Specific PCR
MAIL: Ministry of Agriculture Irrigation and Livestock
MRD: Modified Rogers Distance
SAGA: Genetic Analysis Service for Agriculture
SNP: Single nucleotide polymorphism
