# Supplementary figures and images for: Tracking the adoption of bread wheat varieties in Afghanistan using DNA fingerprinting
Source: BMC Genomics. 2019 Aug 19;20:660. doi: 10.1186/s12864-019-6015-4 (PMC6699131; doi:10.1186/s12864-019-6015-4)

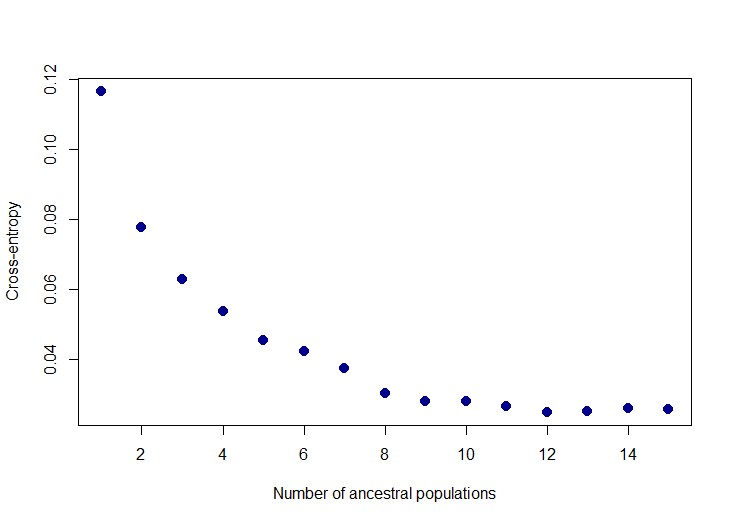

Supplement: Supplementary file 2 — Cross-entropy plot for the SSC when the number of clusters ranges between K = 1–15. (JPG 25 kb) [file 12864_2019_6015_MOESM2_ESM.jpg]

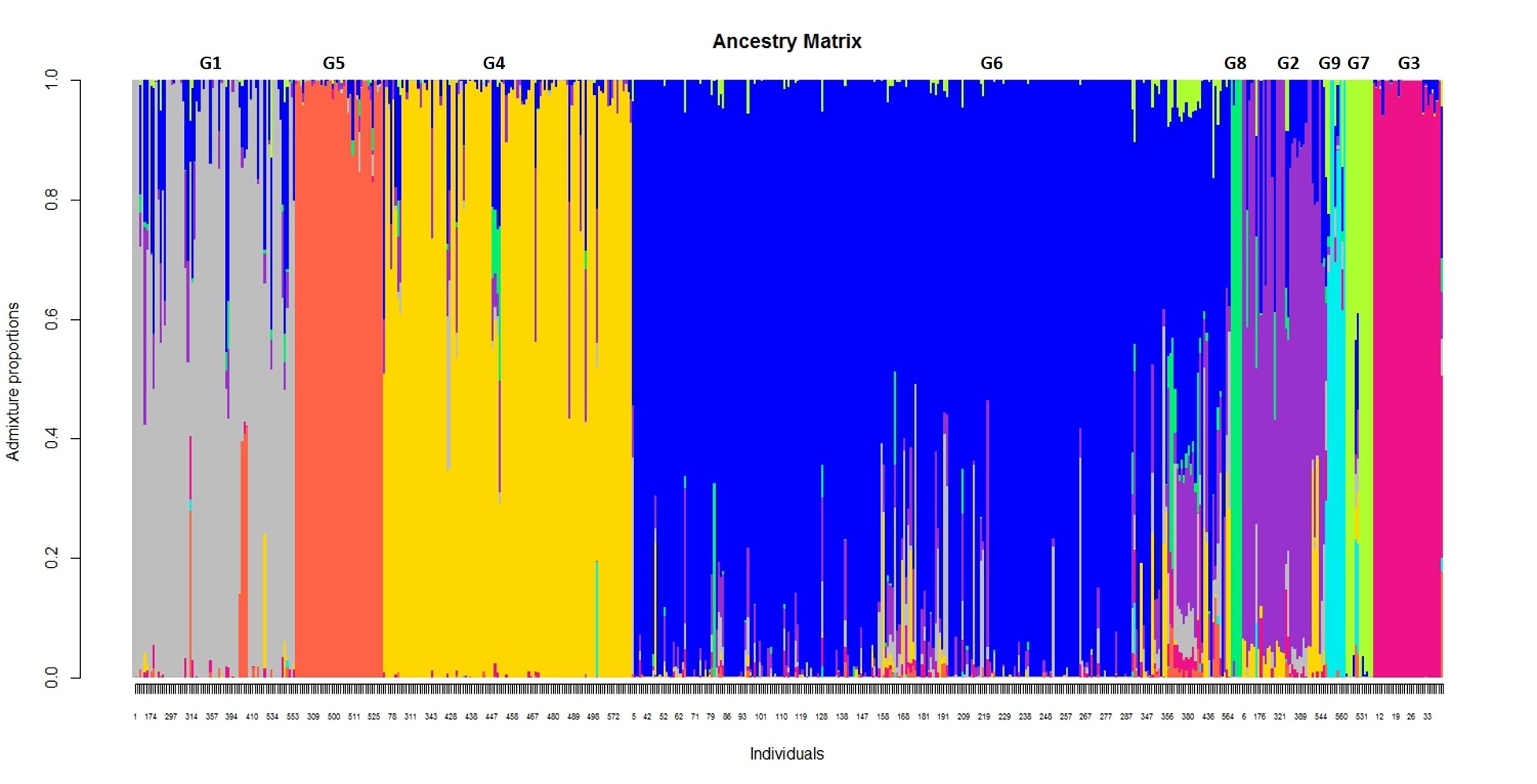

Supplement: Supplementary file 3 — Diagram of groups derived from model based clustering of wheat varieties reported by farmers. (JPG 231 kb) [file 12864_2019_6015_MOESM3_ESM.jpg]
